# 1021- Asthma diagnosis and treatment – 1021. Prevalance of wheezing and risk factor of asthma in preschool children, South Korea

**DOI:** 10.1186/1939-4551-6-S1-P20

**Published:** 2013-04-23

**Authors:** Sung-Il Woo

**Affiliations:** 1 Department of Pediatrics, Chungbuk National University Hispital, Cheongju, South Korea

## Background

Childhood asthma is frequently perceived as a disease with uniform clinical pathways. This perception might be an oversimplification. The aim of the present study was to investigate the prevalence of wheeze in children ≤6 yrs of age and the proportios of children with the risk factors predicting asthma at school children.

## Methods

The Green Breath for Children( GBC) recruited 1,354 preschool children who live in Jincheon area and are ≥2 yrs of age in 2011. Physical examinations, questionare for allergic and respiratory disease and skin prick tests were performed.

## Results

Among 1534 preschool children, 1219 (90.0%) has completed response to questionare. Complete data were available for 1169 children about past medical history including wheeze, familial history and skin prick test. The prevalence of wheeze was 29.4%.It was found that incidence of wheezing declined and incidence of aeroallergen sensitization increased with age. Two hundred and ten preschool children had wheeze at ≥3 yrs of age. Two hundred and seventy three (22.3%) has associated risk factor of progression toward atopic asthma.

## Conclusions

There were high prelevance of wheeze and presence of risk factor of atopic asthma. So it is needed that other methods or markers are able to discriminate children who will have progresssion to asthma and asthma exacerbation in school childhood among wheezer in preschool children.

